# Flavokawain C inhibits proliferation and migration of liver cancer cells through FAK/PI3K/AKT signaling pathway

**DOI:** 10.1007/s00432-024-05639-z

**Published:** 2024-03-09

**Authors:** Rong Wang, Rizhao Li, Huibing Yang, Xuejiao Chen, Liangliang Wu, Xiaohui Zheng, Yuepeng Jin

**Affiliations:** 1 National Key Clinical Specialty (General Surgery), The First Affiliated Hospital of Wenzhou Medical University, Wenzhou, 325000 China; 2 Wenzhou Medical University, Wenzhou, 325000 China

**Keywords:** Flavokawain C, Liver cancer, Proliferation, Migration, FAK/PI3K/AKT pathway, DNA damage

## Abstract

**Purpose:**

This study investigated the potential applicability and the underlying mechanisms of flavokawain C, a natural compound derived from kava extracts, in liver cancer treatment.

**Methods:**

Drug distribution experiment used to demonstrate the preferential tissues enrichment of flavokawain C. Cell proliferation, apoptosis and migration effect of flavokawain C were determined by MTT, colony formation, EdU staining, cell adhesion, transwell, flow cytometry and western blot assay. The mechanism was explored by comet assay, immunofluorescence assay, RNA-seq-based Kyoto encyclopedia of genes and genomes analysis, molecular dynamics, bioinformatics analysis and western blot assay. The anticancer effect of flavokawain C was further confirmed by xenograft tumor model.

**Results:**

The studies first demonstrated the preferential enrichment of flavokawain C within liver tissues in vivo. The findings demonstrated that flavokawain C significantly inhibited proliferation and migration of liver cancer cells, induced cellular apoptosis, and triggered intense DNA damage along with strong DNA damage response. The findings from RNA-seq-based KEGG analysis, molecular dynamics, bioinformatics analysis, and western blot assay mechanistically indicated that treatment with flavokawain C notably suppressed the FAK/PI3K/AKT signaling pathway in liver cancer cells. This effect was attributed to the induction of gene changes and the binding of flavokawain C to the ATP sites of FAK and PI3K, resulting in the inhibition of their phosphorylation. Additionally, flavokawain C also displayed the strong capacity to inhibit Huh-7-derived xenograft tumor growth in mice with minimal adverse effects.

**Conclusions:**

These findings identified that flavokawain C is a promising anticancer agent for liver cancer treatment.

**Supplementary Information:**

The online version contains supplementary material available at 10.1007/s00432-024-05639-z.

## Introduction

Liver cancer stands as a prevailing global malignancy, marked by escalating morbidity and mortality annually (Llovet et al. 2021; Mcglynn et al. 2021). The nonspecific nature of early liver cancer symptoms, coupled with the absence of effective early screening methods, contributes to delayed-stage diagnosis, thereby engendering treatment complexities. Furthermore, the high metabolic rate and the swift drug clearance, along with drug resistance and drug-induced liver damage, curtailed the effectiveness of conventional treatment modalities, including surgery, chemotherapy, and radiotherapy (Anwanwan et al. 2020; Sperandio et al. 2022). Furthermore, the intricate interplay of liver cancer's heterogeneity, complexity, and immune tolerance undermines the feasibility of achieving clinical expectations through targeted therapy and immunotherapy (Huang et al. 2020; Llovet et al. 2022; Sperandio et al. 2022). Additionally, the pronounced aggressiveness and the propensity for easy metastasis of liver cancer cells contribute significantly to the poor prognosis of liver cancer patients (Anwanwan et al. 2020). In light of the aforementioned factors, despite the swift advancement of medical expertise over the previous decade, considerable drawbacks and constraints persist in the clinical management of liver cancer. Consequently, there exists a pressing need for novel therapeutic interventions and/or more efficient pharmaceutical agents in the management of liver cancer.

Natural products have consistently been regarded as a crucial reservoir for novel drug discovery (Chopra et al. 2021; Orhan 2022; Raslan 2022; Zhang et al. 2022), particularly in the realm of anticancer drug development (Varghese et al. 2021; Naeem et al. 2022). Among these natural compounds, flavokawain derivatives have been documented to possess notable anticancer activities against a spectrum of cancer cells (Teschke et al. 2011; Abu et al. 2013; Wang et al. 2021). Notably, flavokawain A and B have demonstrated significant anticancer efficacy across a range of malignancies including breast cancer (Abu et al. 2015, 2016), lung cancer (Hseu et al. 2019; Li et al. 2020), bladder cancer (Liu et al. 2017), liver cancer (Pinner et al. 2016), and melanoma (Hseu et al. 2020a, b). Furthermore, the fundamental anticancer mechanisms underlying flavokawain A and B primarily center around the ROS signaling pathway (Pinner et al. 2016; Chang et al. 2017; Hseu et al. 2019; Hseu et al. 2020a, b; Hseu et al. 2020a, b), the mTOR signaling pathway (Liu et al. 2017), and the PI3K/AKT signaling pathway (Hua et al. 2020). In contrast to flavokawain A and B, limited research has shown that flavokawain C exhibits anticancer effects specifically against colon cancer (Phang et al. 2017, 2021) and breast cancer (Lin et al. 2023). The potential of flavokawain C as a prospective drug for liver cancer treatment, along with its associated anticancer mechanisms, remains uncertain and warrants thorough investigation.

In the present study, findings revealed that flavokawain C exhibited a predilection for in vivo accumulation in liver tissues with minimal impact on normal liver tissue, and concurrently exerted substantial inhibitory effects on the proliferation and migration of liver cancer cells in vitro and in vivo by downregulating the FAK/PI3K/AKT pathway. These results underscored the promising potential of flavokawain C as a targeted therapeutic agent for liver cancer treatment.

## Materials and methods

### Cells culture

Liver cancer cell lines (Huh-7, Hep3B and HepG2) and normal liver cell line (MIHA) were obtained from the Chinese Academy of Sciences (CAS) and the Shanghai Academy of Biological Sciences (SABS), respectively. Huh-7 and Hep3B cells were cultured in DMEM (Gibco, USA) containing 10% FBS (PPA-GE, USA) and penicillin/streptomycin (100 U/mL). HepG2 cells were maintained in MEM (Sigma-Aldrich, USA) with 10% FBS (PPA-GE, USA) and penicillin/streptomycin (100 U/mL). MIHA cells were cultured in DMEM (Gibco, USA) containing 15% FBS (PPA-GE, USA) and penicillin/streptomycin (100 U/mL). The cell lines were maintained in a cell incubator under 37 °C with 5% CO_2_.

### Reagents and antibodies

Flavokawain C (Catalog No. PHL83854) was purchased from sigma-Aldrich with a minimum purity of 98%. Serum alanine transferase (ALT) assay kit (Catalog No. C009-2-1) and serum aspartate aminotransferase (AST) assay kit (Catalog No. C010-2-1) were obtained from Nanjing Jiancheng Bioengineering Institute. Antibodies against Bcl2 (Catalog No. sc-7382) and Bax (Catalog No. sc-7480) were acquired from Santa Cruz Biotechnology. Antibodies against p-PI3K (Catalog No. AF3242) and PI3K (Catalog No. AF1549) were obtained from Affinity Biosciences. Antibodies against p-AKT (Catalog No. 66444-1-Ig) and GAPDH (Catalog No. 60004-1-Ig) were purchased from Proteintech. Antibodies against γ-H2AX (Catalog No. 9718), AKT (Catalog No. 4685), FAK (Catalog No. 71433), and phosphor-FAK (Catalog No. 8556) were obtained from Cell Signaling Technology. Anti-mouse lgG, HRP-linked antibody (Catalog No. 7076S) was purchased from Cell Signaling Technology.

### Animal preparation and ethics statement

Kunming mice (KM) and BALB/c nude mice were procured from Beijing Vital River Laboratory Animal Technology Co., Ltd, located in Beijing, China. The mice were housed in pathogen-free facilities maintained at a controlled temperature of 22–23 °C. Adequate food and water were provided, and the animals were cared for in strict adherence to the guidelines outlined in the Guide for the Care and Use of Laboratory Animals. Ethical clearance for all animal-related experiments was obtained from the Animal Care and Use Committee of Wenzhou Medical University (Approval Document No. wydw 2023-0294).

### Tissue distribution study

Twenty KM mice were randomly divided into two groups, each containing ten mice. The mice were orally administered either 20 mg/kg of flavokawain C (formulated in 10% Tween 80, 10% ethanol, and 80% saline) or normal saline. The mice were euthanized 2 h after receiving flavokawain C or normal saline. Tissues were collected, washed in normal saline, homogenized, and prepared for sampling. Subsequently, the concentration of flavokawain C was determined using UHPLC-MS/MS.

### ALT and AST determination assay

Forty KM mice were randomly divided into four groups and orally administered the specified doses of flavokawain C (10, 30, 90 mg/kg) or normal saline (Control). Blood samples were collected from the ocular region 7 days after the administration of flavokawain C (formulated in 10% Tween 80, 10% ethanol, and 80% saline) or normal saline. ALT and AST activities were assessed according to the manufacturer's guidelines. Briefly, blood was centrifuged, and the resulting supernatant was collected. Substrate solution (20 μL per well) and sample (5 μL per well) were added to a 96-well plate and incubated at 37 °C for 30 min. Subsequently, 2,4-dinitrophenylhydrazine solution (20 μL per well) and sample (5 μL per well) were added and incubated for an extra 20 min. Lastly, 0.4 mol/L sodium hydroxide solution (200 μL per well) was added, and the plate was maintained at room temperature for 15 min before absorbance measurement.

### Hematoxylin eosin (H&E) staining assay

The H&E staining assay was conducted following the previously described method (Shen et al. 2022). In brief, liver tissue or tumor tissue samples were fixed with 4% paraformaldehyde (Beyotime, China), embedded in paraffin, and then cut into 5 mm-thick sections. Subsequently, the sections were stained with H&E and visualized using a Nikon Ti microscope (Nikon, Japan).

### Immunohistochemistry (IHC) assay

Summarily, tumors were fixed in 4% paraformaldehyde for 48 h. Subsequently, the tumors were meticulously processed, embedded in paraffin, and sectioned into 5 µm slices. After baking for 4–5 h followed by dewaxing, a subset of histological sections underwent IHC analysis, the Mouse anti-Ki67 monoclonal antibody (dilution 1:50, SANTA, China), and the Mouse anti-γ-H2AX monoclonal antibody (dilution 1:150, Millipore, USA) were incubated overnight at 4 °C. Subsequently, the sections were treated with an HRP-conjugated secondary antibody at room temperature for 1 h. Finally, the sections were stained with DAB (ZSGB-BIO, China) and counterstained with hematoxylin for enhanced visibility and analysis.

### MTT assay

Huh-7, HepG2, Hep3B, and MIHA cells were seeded in 96-well plates (Corning, USA) at a density of approximately 6 × 10^3^ cells per well. The cells were incubated at 37 °C for 48 h, both with and without treatment of flavokawain C. Subsequently, MTT solution (0.5 mg/mL, 20 μL/well) was added and incubated for 4 h. After removing the culture medium, 100 μL of dimethyl sulfoxide (DMSO) was added to dissolve the formazan reaction product. The absorbance of the mixture was measured at 490 nm using a DTX880 spectrophotometer (Beckman Coulter, USA) to assess cell viability.

### Colony-formation assay

Huh-7, HepG2, Hep3B, and MIHA cells were seeded in 12-well plates at a density of 800 cells per well. Following attachment, the cells were incubated for 2 weeks in regular growth media with 0.01% DMSO (Control) or co-incubated with the specified concentrations of flavokavain C. The colonies were subsequently washed three times with PBS, fixed with 4% formaldehyde for 15 min, and stained with 0.04% crystal violet for 1 h. The colonies on each plate were observed under a microscope after being washed twice with ddH_2_O. The colony count was ascertained in three independent experiments.

### EdU staining assay

The EdU staining assay was performed according to the manufacturer's guidelines. Briefly, Huh-7 and Hep3B cells were transfected and seeded in 12-well plates containing coverslip at a density of 5 × 10^4^ cells per well. The cells then incubated overnight. Subsequently, the cells were exposed to either 0.01% DMSO (Control) or the specified concentrations of flavokawain C for 48 h. Lastly, cell proliferation was evaluated using the EdU staining proliferation kit (Beyotime, China), and the resulting stained cells were visualized under a Nikon fluorescence microscope.

### Flow cytometry apoptosis assay

Apoptotic cell was identified using an apoptosis detection kit from BD Biosciences (USA). Huh-7 and Hep3B cells were incubated with either 0.01% DMSO (Control) or varying concentrations of flavokawain C (4, 8, and 16 μM) for 48 h. The cells were then stained with Annexin V and propidium iodide (PI) for 15 min. After that, Annexin V binding buffer was added to the mixture, and fluorescence was determined using a FACSC flow cytometer (BD Biosciences). The obtained data was analyzed with Flowjo 9.0 software.

### Western blot assay

Cells were cultured in 6-well plates (Corning, USA) and then exposed to the specified concentrations of flavokawain C for 48 h. Subsequently, the cells were lysed, and total protein was extracted and quantified through the Bradford assay. The protein samples were electrophoresed on 10% SDS-PAGE gels and subsequently transferred to PVDF membranes (Bio-Rad). These membranes were then blocked with a 5% skim milk solution in TBST buffer and subsequently incubated with primary antibodies. After washing the PVDF membranes with TBST, an HRP-conjugated secondary antibody (CST, Danvers, MA) was applied. The immune-reactive bands were visualized by employing an enhanced chemiluminescence (ECC) reagent (Bio-Rad, USA).

### Alkaline comet assay

The alkaline comet assay was performed as described method in a previous study (Shen et al. 2022). Following a 48 h treatment with either 0.01% DMSO (Control) or flavokawain C at concentrations of 4, 8, and 16 μM, cells were harvested and then placed onto slides coated with a mixture of 1.5% normal agarose and 0.5% low-melting temperature agarose. Next, the slides were subjected to lysis using a buffer comprising 0.5% Triton X-100, 2.5 M NaCl, 10 mM Tris (pH 10.0), 3% DMSO, 100 mM EDTA, and 1% *N*-lauroylsarcosine. Electrophoresis was carried out at 1.5 V/cm in Tris–HCl (100 mM), 1% DMSO, and 300 mM sodium acetate buffer for 20 min. After electrophoresis, the slides were counter-stained with a solution of propidium iodide (PI) at a concentration of 0.02 mg/mL and observed under a fluorescence microscope (Nikon, Japan).

### Immunofluorescence (IF) assay

The IF assay was performed as described method in a previous study (Qiu et al. 2022). Briefly, cells were seeded onto a coverslip (Corning, USA) and then fixed with 4% paraformaldehyde (Beyotime, China) for 15 min at room temperature. Subsequently, the cells were permeabilized with 0.5% Triton X-100 for 30 min. Following washing, the coverslip was incubated in a blocking solution for 1 h. Primary antibodies (53BP1 or FAK) were applied and allowed to incubate overnight. After three washes with PBST, the cells were incubated with DyLight 488-conjugated secondary antibodies for another 1.5 h. Following another round of washing with PBST, the coverslip was stained with DAPI (Beyotime, China) and then examined using a fluorescence microscope (Nikon, Japan).

### Cell adhesion assay

The cell adhesion assay was performed as described method in a previous study (Qiu et al. 2022). Briefly, human fibronectin (2.5 mg/mL) was dissolved in PBS (Millipore, CA) and utilized to coat a 96-well plate overnight at 4 °C. Cells were then seeded into each well at a density of 5 × 10^4^ cells per well in serum-free medium. The plate was subsequently placed in a CO_2_ incubator at 37 °C for cell cultivation. Following cell attachment, the medium was removed, and the cells were fixed using 4% paraformaldehyde. Subsequently, the cells were stained with crystal violet for 5 min at room temperature. The crystal violet was then dissolved in 100 mL of 33% acetic acid, and the absorbance was measured at 560 nm using a Multiskan FC automatic microplate reader (Thermo Fisher, USA). The relative number of cells adhering to the extracellular matrix was calculated using the following equation: the mean optical density (OD) of treated cells divided by the mean OD of control cells. Cells treated with a vehicle (0.1% DMSO) was used as the control.

### Trans-well assay

The trans-well assay was conducted employing 24-well trans-well chambers (Corning Costar, NY) following the guidelines provided by the manufacturer. In brief, cells were cultured with either 0.01% DMSO (Control) or different concentrations of flavokawain C (4, 8, 16 μM) for 48 h. Then, a prepared cell suspension in FBS-free DMEM (100 μL) was introduced into the upper chamber at a density of 1 × 10^5^ cells/well. In the lower chamber, 600 µL of DMEM containing 10% FBS was added. After 48 h, the lower surface of the chamber was fixed using 4% paraformaldehyde for 15 min at room temperature. Subsequently, the cells were stained with a 0.1% crystal violet solution for 5 min at room temperature. Photomicrographs were captured using a Nikon microscope. The crystal violet dye was then dissolved in 500 μL of 33% acetic acid, and the absorbance was subsequently measured at 560 nm.

### RNA sequencing assay

Huh-7 cells underwent treatment with either 0.01% DMSO or 16 μM flavokawain C for 48 h. Afterward, the cells were harvested, and total RNA extraction was performed utilizing TRIzol reagent (Invitrogen, CA, USA). Paired-end sequencing was conducted at LC-BIO (Hangzhou, China) using an Illumina HiSeq 4000, following the vendor’s recommended protocol.

### Molecular docking (MD) and simulations

The crystal structures for PI3K (PDB ID: 4ZOP) and FAK (PDB ID: 4GU9) were obtained from the Protein Data Bank. Molecular docking simulations were performed employing SYBYL-X 2.0 software, following the methodology described previously (Liang et al. 2021). Initially, the protein's PDB structure was acquired from the official website, and any solvent molecules and extraneous entities were removed. Subsequently, the molecular structure was optimized through energy minimization calculations. Processed protein and small molecule ligand were subjected to docking simulations, and the conformation with the highest score and the lowest energy was chosen. The resulting complex was analyzed for visualization using PyMOL. The MD simulations were carried out using Amber 20. The protein was prepared using the ff14SB force field, and the ligand was prepared using the GAFF force field with AM1-BCC charge. The MD simulation system was built using the OPC solvent model with a box shape of truncated octahedron and a margin distance of 10 Å (FAK-FKC) or 12 Å (PI3K-FKC). All systems were neutralized with explicit counterions (Na^+^ or Cl^−^). Simulations were run 10 ns under the condition of 300 K and 1 atm by applying periodic boundary conditions.

### The Cancer Genome Atlas (TCGA) analysis

TCGA analysis was carried out using TTCGA database available at (http://cancergenome.nih.gov/). Differential expression analysis was executed using R language (version 4.0.2) and GraphPad Prism 5.0.

### Tumor xenograft model

The Laboratory Animal Resources Center of Wenzhou Medical University approved all animal experiments (Date: June 08, 2023/No: wydw2023-0294). The tumor xenograft model was conducted using 6-week-old BALB/c nude mice. Huh-7 cells (1 × 10^6^) in PBS were subcutaneously injected into the right flank of BALB/c nude mice. Initially, six mice with good health were assigned to each group after five days of modeling. After ten days of modeling, the average tumor volume went to about 200 mm^3^, and some mice were excluded for the significant variations in tumor volumes, resulted in four mice per group ultimately. Then flavokawain C (16 mg/kg), which was formulated in 10% Tween 80, 10% ethanol, and 80% saline, injected intraperitoneally daily for 2 weeks. Volume of the tumor was measured every two days using calipers, and volume was calculated using *V* = ab^2^/2, where a and b are the length and width of the tumor, respectively.

### Statistical analysis

Values were presented as mean ± SD. Statistical analysis was conducted using GraphPad Prism 5.0 software. Group comparisons were performed using a two-tailed, unpaired Student's *t* test to evaluate intergroup variations. Statistical significance was defined at a significance level of *p* < 0.05 (*p < 0.05, **p < 0.01, ***p < 0.001).

## Results

### Flavokawain C preferred to accumulated in liver tissues in vivo with minimal side-effect

**Fig. 1:**
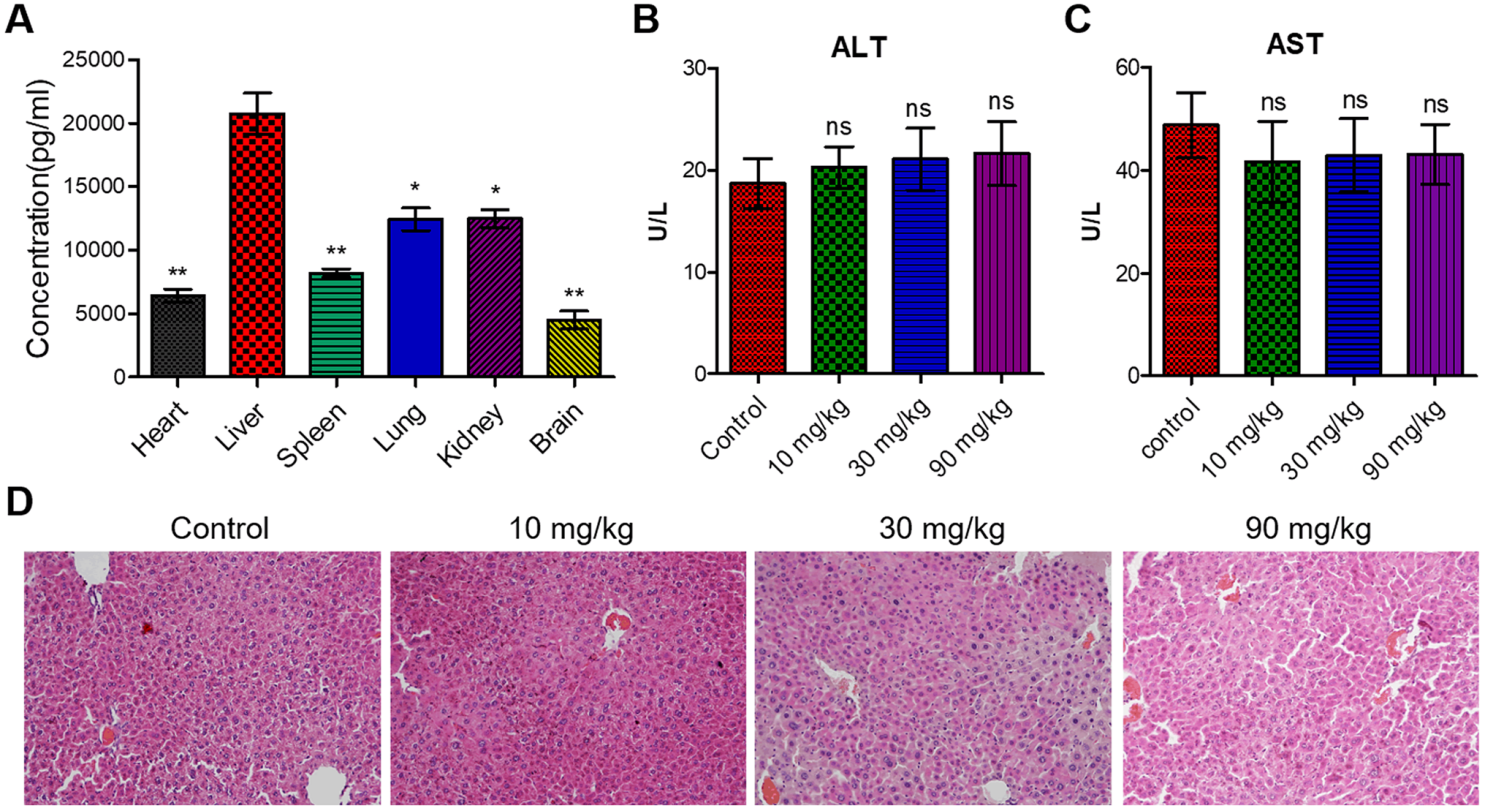
Flavokawain C preferentially enriches in liver tissues and has minimal impact on normal liver in vivo.** A** In vivo distribution of flavokawain C. Ten mice were orally administered 20 mg/kg of flavokawain C or normal saline and euthanized after 2 h. **B**, **C** Serum ALT and AST concentrations in mice treated with flavokawain C or normal saline. Forty mice were randomly divided into four groups and orally given the specified doses of flavokawain C (10, 30, 90 mg/kg) or normal saline (Control). Blood samples were collected from the ocular region after administration of flavokawain C or normal saline for 7 days. **D** H&E staining assay of liver tissue sections from flavokawain C-treated or -untreated mice. The mice were treated as B or C. The results were derived from three independent experiments, and the values were expressed as mean ± SD. Statistical analysis utilized a two-tailed, unpaired Student's *t*-test (ns indicates no significance with *p* > 0.05, *p* < 0.05, *p < 0.01, **p < 0.001)

The results of the in vivo drug distribution experiment demonstrated predominant accumulation of flavokawain C in the liver tissue of mice following oral administration (Fig. 1A). In additional to targeting tumor tissues, hepatotoxicity poses a significant challenge in the development and utilization of therapy drug for liver cancer (Mahmoudi et al. 2023; Vigano et al. 2023), particularly those derived from herbal products (Lee et al. 2015; He et al. 2019). Accordingly, the potential impact of flavokawain C on liver function was assessed through quantification of serum levels of alanine transaminase (ALT) and aspartate transaminase (AST) (Juan et al. 2023). The results indicated that treatment with flavokawain C did not lead to alterations in serum levels of ALT and AST (Fig. 1B, C), demonstrating flavokawain C has no impact on liver function. Additionally, the H&E staining assay demonstrated that flavokawain C treatment did not induce changes in liver tissue structure (Fig. 1D). Moreover, the MTT assay revealed substantial anticancer potential of flavokawain C against liver cancer Huh-7, Hep3B, and HepG2 cells, with the half inhibitory concentration (*IC*_50_) values were 23.42 ± 0.89 µM, 28.88 ± 2.60 µM, and 30.71 ± 1.27 µM (Table S1), respectively. In contrast, flavokawain C demonstrated reduced toxicity toward normal liver MIHA cells compared to liver cancer cells, displaying an *IC*_50_ value of 53.95 ± 5.08 µM (Table S1). Collectively, these findings strongly indicate the considerable therapeutic potential of flavokawain C for liver cancer treatment.

### Flavokawain C selectively inhibited liver cancer cell proliferation and induced cell apoptosis

**Fig. 2:**
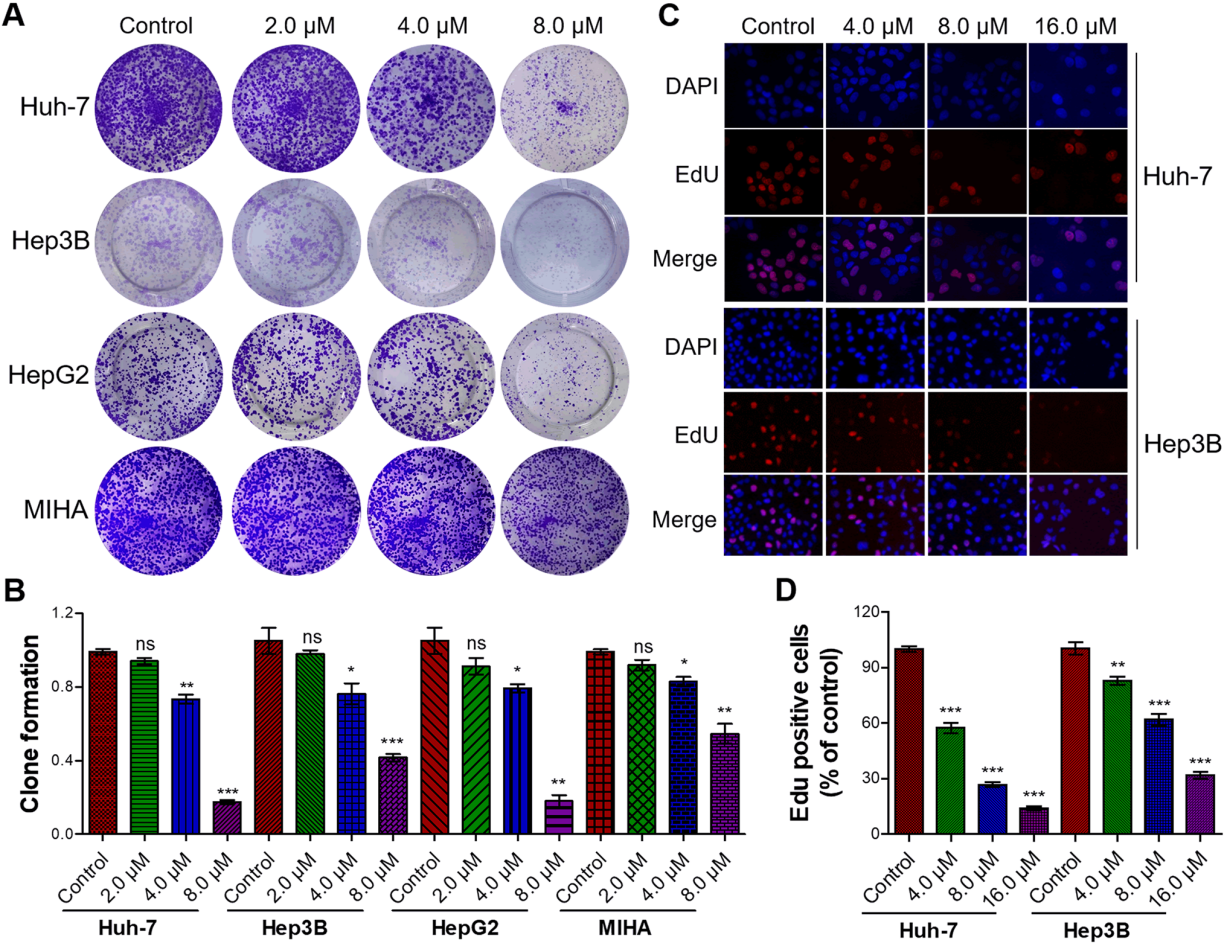
Flavokawain C inhibits the colony formation and DNA replication of liver cancer cells. **A** Flavokawain C suppresses colony formation in liver cancer cells (Huh-7, Hep3B, and HepG2), with a weaker impact on normal liver MIHA cells. Cells were incubated for 2 weeks with indicated concentrations of flavokawain C treatment, while 0.01% DMSO was utilized as the control. **B** Quantitative analysis of A. **C** Representative images from the EdU staining assay indicating flavokawain C-mediated inhibition of DNA replication in liver cancer cells Huh-7 and Hep3B in a dose-dependent manner. Cells were treated with the indicated concentration of flavokawain C or 0.01% DMSO (Control) for 48 h prior to the EdU staining assay. **D** Quantitative analysis of C. The results were derived from three independent experiments and were presented as mean ± SD. The statistical analysis was conducted using a two-tailed, unpaired Student's *t* test (ns means no significance with *p* > 0.05, *p < 0.05, **p < 0.01, ***p < 0.001)

To further investigate the anticancer potential of flavokawain C in liver cancer cells, a colony-formation assay was initially performed. The results demonstrated that treatment with flavokawain C suppressed colony formation in liver cancer cells, while exhibiting a weaker inhibitory effect on normal liver cells at equivalent treatment concentrations (Fig. 2A). Subsequent quantitative analysis revealed that flavokawain C treatment dose-dependently inhibited colony formation of liver cancer cells (Huh-7, Hep3B and HepG2) and normal liver MIHA cells (Fig. 2B). Notably, the inhibitory impact on liver cancer cells significantly exceeded that observed in normal liver cells (Fig. 2B). Concurrently, the result of the EdU staining assay indicated that treatment with flavokawain C dose-dependently suppressed the DNA replication rate in liver cancer Huh-7 and Hep3B cells (Fig. 2C, D).

**Fig. 3:**
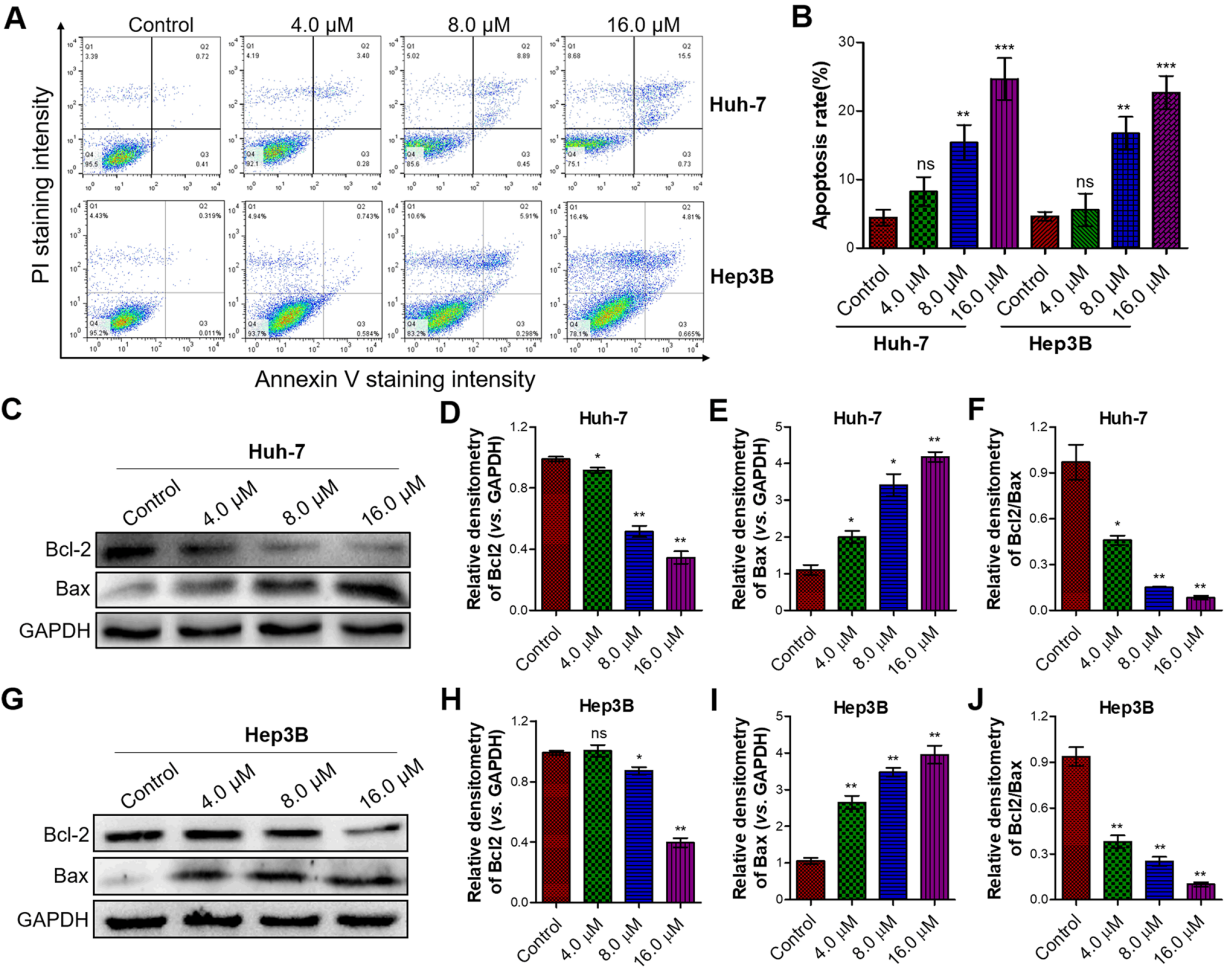
Flavokawain C induces apoptosis in liver cancer cells. **A** Flow cytometry analysis assessed apoptosis in liver cancer cells (Huh-7 and Hep3B) with or without flavokawain C treatment. Cells were treated with the specified concentration of flavokawain C or 0.01% DMSO (Control) for 48 h prior to the flow cytometry assay. **B** Quantitative analysis of A. **C** Western blot analysis assessed the levels of the anti-apoptotic protein Bcl2 and the pro-apoptotic protein Bax in liver cancer cells (Huh-7) with or without flavokawain C treatment. Cells were treated as described in **A**. **D–F** Quantitative analysis of C. **G** The same as C, except Hep3B cells were used. **H–J** Quantitative analysis of G. The results were derived from three independent experiments and the values were presented as mean ± SD. The statistical analysis was conducted using a two-tailed, unpaired Student's *t* test (ns means no significance with *p* > 0.05, *p < 0.05, **p < 0.01, ***p < 0.001)

To further investigate the impact of flavokawain C treatment on the fate of liver cancer cells, flow cytometry using dual staining with PI and annexin V were performed. Flow cytometry results indicated that flavokawain C treatment induced cell apoptosis (Fig. 3A). Moreover, quantitative analysis revealed a positive correlation between the treatment concentration of flavokawain C and the proportion of apoptotic cells (Fig. 3B). Additionally, the western blot assay indicated a dose-dependent reduction in the levels of the anti-apoptotic protein Bcl2 (Tsujimoto 1998) and a marked elevation in the levels of the pro-apoptotic protein Bax (Er et al. 2006) upon flavokawain C treatment in liver cancer Huh-7 cells (Fig. 3C–F). Similar experimental observations were also noted in liver cancer Hep3B cells treated with flavokawain C (Fig. 3G–J). Collectively, these findings illustrated that flavokawain C promoted apoptosis in liver cancer cells through concurrent modulation of Bcl2 and Bax protein expression.

### Flavokawain C induced intense DNA damage and provoked strong DNA damage response in liver cancer cell

**Fig. 4:**
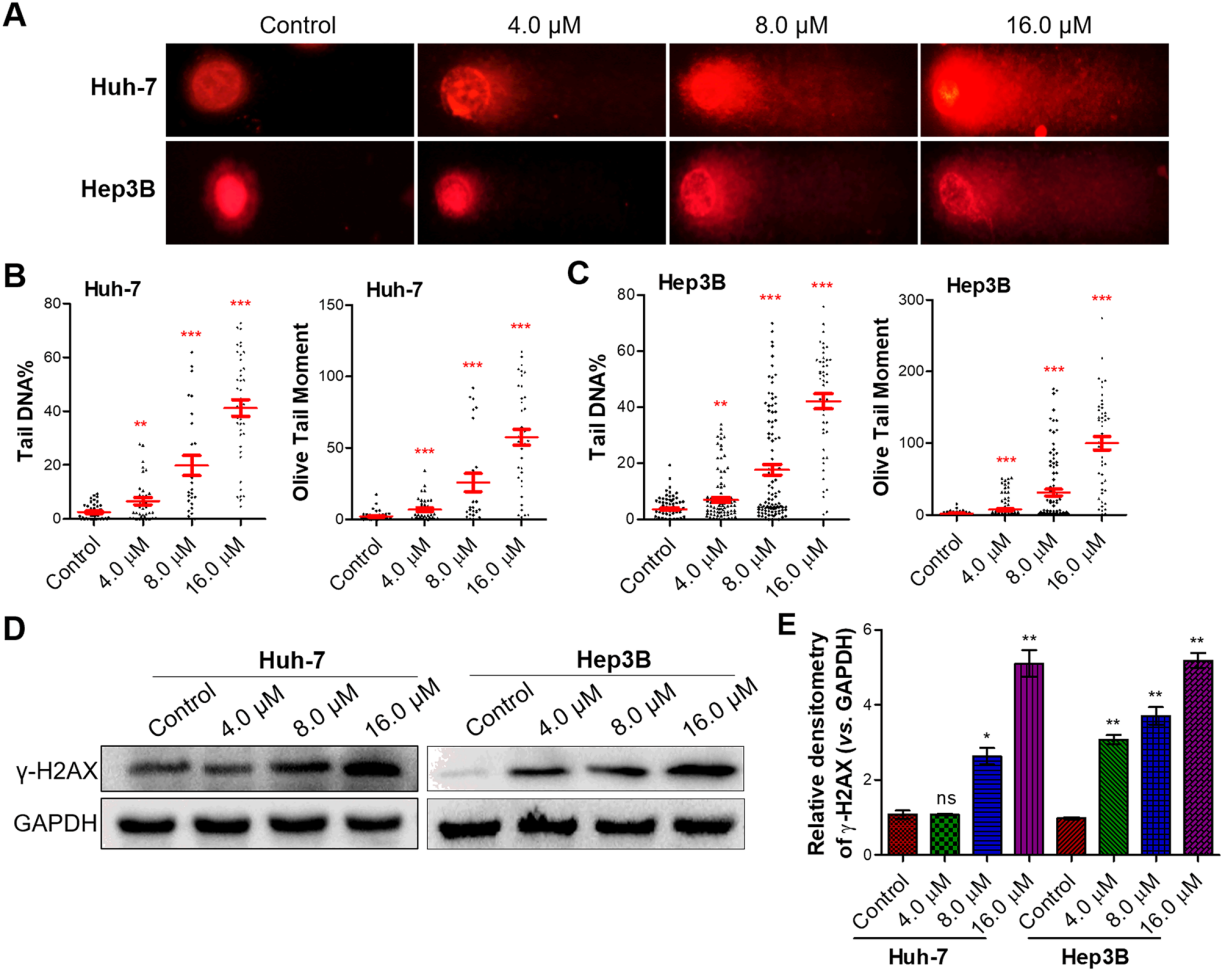
Flavokawain C induces DNA damage in liver cancer cells. **A** Alkaline comet assay assessing DNA damage induced by flavokawain C in liver cancer cells (Huh-7 and Hep3B). Cells were treated with the specified concentration of flavokawain C or 0.01% DMSO (Control) for 48 h before being subjected to the comet assay. **B**, **C** Quantitative analysis of A. **D** Western blot assay determined the abundance of γ-H2AX in liver cancer cells (Huh-7 and Hep3B) treated with or without flavokawain C. Cells were treated as A. GAPDH was used as a positive control. **E** Quantitative analysis of D. The results were obtained from three independent experiments and the values were presented as mean ± SD. The statistical analysis was conducted using a two-tailed, unpaired Student's *t*-test (ns means no significance with *p* > 0.05, *p < 0.05, **p < 0.01, ***p < 0.001)

Given that DNA constitutes the principal target of most natural anticancer agents (Wang et al. 2013), we hypothesized that the potential anticancer mechanism of flavokawain C might involve DNA mediation. To assess the extent of DNA damage in liver cancer Huh-7 and Hep3B cells in response to flavokawain C treatment, the alkaline comet assay was conducted (Olive et al. 2006). In the comet assay, damaged DNA would migrate from the nucleus to create a comet-like tail (Olive et al. 2006). The results demonstrated a notable dose-dependent elevation in the comet tail intensity in liver cancer Huh-7 and Hep3B cells upon flavokawain C treatment, indicating that flavokawain C could induce DNA damage (Fig. 4A). Consistently, the percentage of tail DNA, acquired from tail length and DNA quantification in the tail, indicated an incremental rise in tail DNA% in both Huh-7 and Hep3B cells due to flavokawain C treatment, further supporting the notion of flavokawain C-triggered intense DNA damage (Fig. 4B, C). In addition to tail DNA%, the olive tail moment, which gages both the DNA quantity and distribution within the tail, also confirmed the substantial DNA damage resulting from flavokawain C treatment (Fig. 4B, C).

**Fig. 5:**
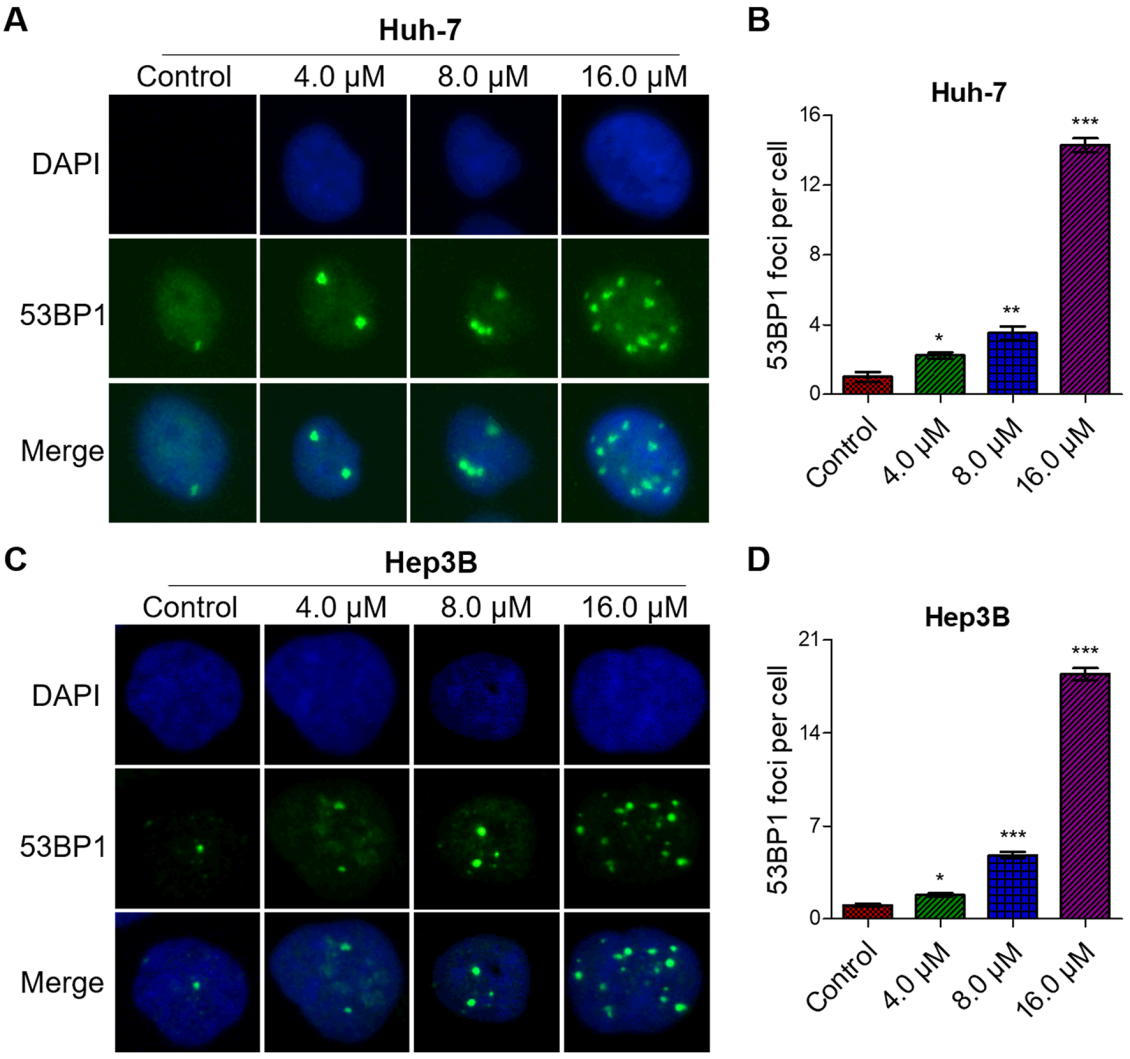
Flavokawain C provokes DNA damage response in liver cancer cells. **A**, **C** Representative images from the immunofluorescence (IF) assay demonstrating flavokawain C-induced strong DNA damage response. Cells were treated with the specified concentration of flavokawain C or 0.01% DMSO (Control) for 48 h before being subjected to the IF assay. The marker 53BP1 (green) was used to indicate DNA damage response, while DAPI (blue) labeled the nuclei. **B**, **D** Quantitative analysis of A and C, respectively. The results were obtained from three independent experiments and the values were presented as mean ± SD. The statistical analysis was conducted using a two-tailed, unpaired Student's *t*-test (*p < 0.05, **p < 0.01, ***p < 0.001)

DNA damage triggers a DNA damage response, detectable through the classic markers of DNA damage response: γ-H2AX or 53BP1 (Williamson et al. 2020). The western blot assay demonstrated that flavokawain C treatment significantly increased the abundance of γ-H2AX in both Huh-7 and Hep3B cells (Fig. 4D). Quantitative analysis revealed that the abundance of γ-H2AX increased in a dose-dependent manner upon flavokawain C treatment (Fig. 4E). Consistent with this, IF assay demonstrated a significant dose-dependent increase in the number of 53BP1 foci upon flavokawain C treatment in both Huh-7 and Hep3B cells (Fig. 5A, C). Quantitative analysis indicated that the high treatment concentration (16.0 μM) of flavokawain C would induce over 15 53BP1 foci per cell, in contrast to the control group with an average of 1.5 foci per cell (Fig. 5B, D). Taken together, these findings strongly suggest that flavokawain C treatment induces substantial DNA damage and triggers a robust DNA damage response in Huh-7 and Hep3B liver cancer cells.

### Flavokawain C weakened cell-extracellular adhesion and inhibited cell migration of liver cancer cell

**Fig. 6:**
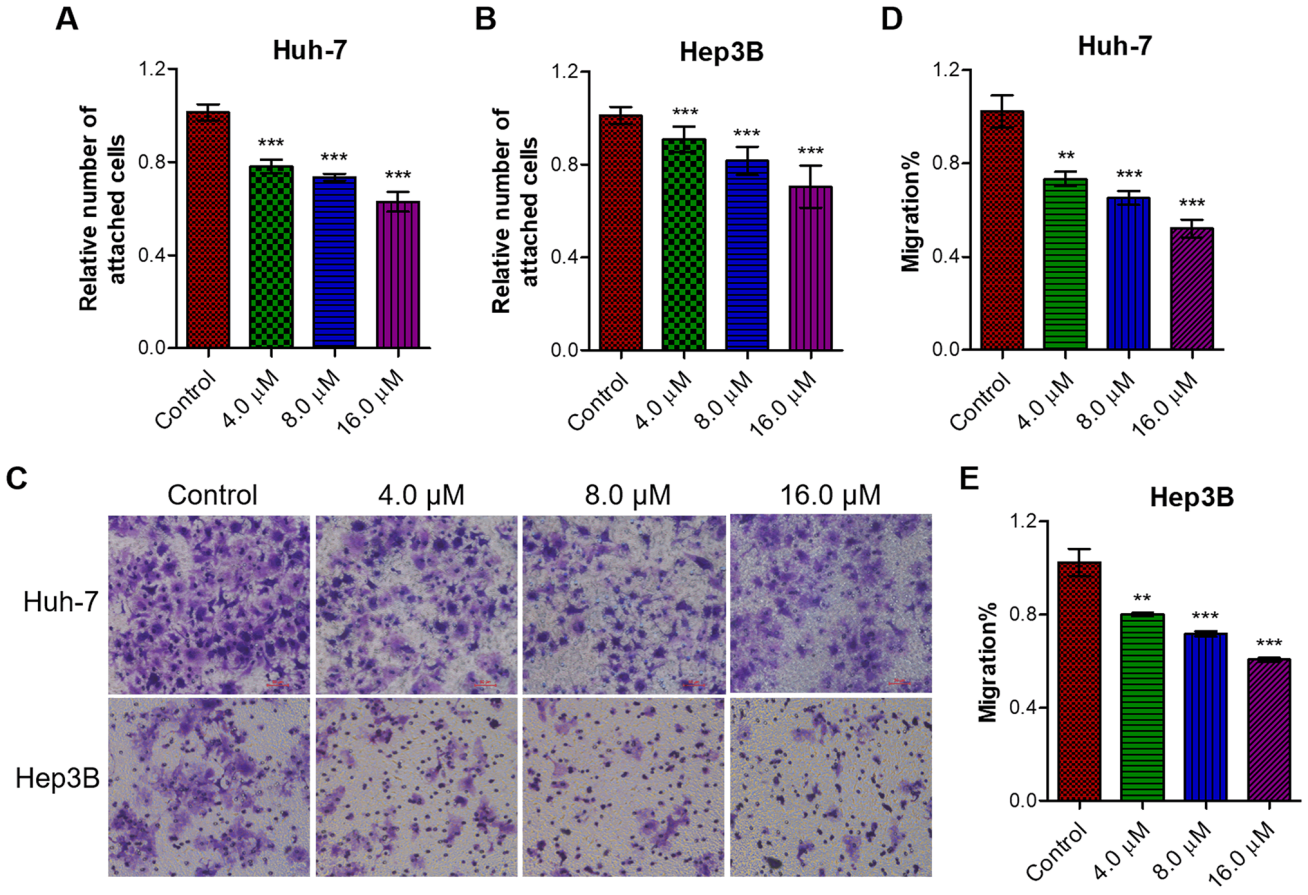
Flavokawain C inhibits the cell–matrix adhesion and migration of liver cancer cells. **A** Cell adhesion assay assessed the impact of flavokawain C treatment on cell–matrix adhesion of Huh-7 cells. Cells were treated with the specified concentration of flavokawain C or 0.01% DMSO (Control) for 48 h prior to the cell adhesion assay. **B** The same as **A**, except Hep3B cells were used. **C** Trans-well assay determined the migration ability of liver cancer cells (Huh-7 and Hep3B). Cells were treated as A. **D**, **E** Quantitative analysis of C. The results were obtained from three independent experiments and the values were presented as mean ± SD. The statistical analysis was conducted using a two-tailed, unpaired Student's *t*-test (**p < 0.01, ***p < 0.001)

To further investigate the impact of flavokawain C on the migration of liver cancer cells, cell adhesion assay and trans-well assay were conducted. The results of the cell adhesion assay demonstrated that treatment with flavokawain C dose-dependently reduced the adhesion ability of Huh-7 cells to the extracellular matrix (Fig. 6A). A similar experimental phenomenon was observed in Hep3B cells, where the group treated with flavokawain C exhibited weaker cell-extracellular matrix adhesion compared to the control group (Fig. 6B). Furthermore, the results of the trans-well assay indicated that compared to the untreated Huh-7 and Hep3B cells (control group), cells treated with flavokawain C exhibited reduced membrane penetration ability, resulting in fewer migrating cells through the membrane (Fig. 6C). Corresponding quantitative analysis demonstrated that flavokawain C dose-dependently inhibited the migration of Huh-7 and Hep3B cells (Fig. 6D, E). Treatment with a high concentration (16 μM) of flavokawain C resulted in an inhibition of approximately 50% of cell migration (Fig. 6D, E).

### Flavokawain C down-regulated the FAK/PI3K/AKT signaling pathway

**Fig. 7:**
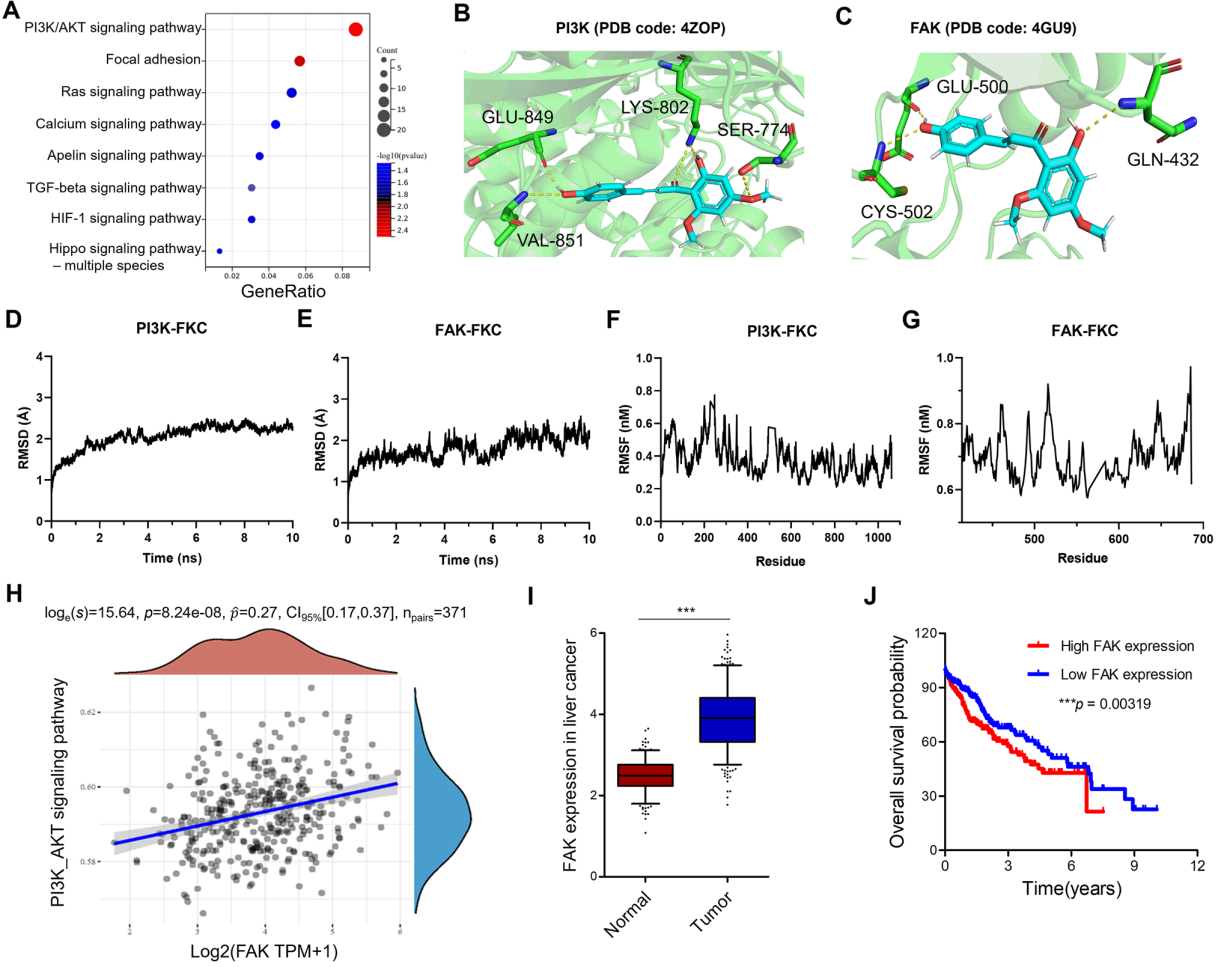
Flavokawain C regulates FAK/PI3K/AKT signaling pathway. **A** RNA-seq-based KEGG pathway analysis of differentially regulated pathways in Huh-7 cells treated with 16.0 µM flavokawain C for 48 h compared to the control (0.01% DMSO). **B**, **C** Molecular docking identified the binding sites and modes between flavokawain C and PI3K or FAK, respectively. **D**, **E** RMSD of the PI3K–flavokawain C and the FAK–flavokawain C complex. **F**, **G** RMSF of the PI3K–flavokawain C and the FAK–flavokawain C complex. **H** Bioinformatics analysis suggested a positive relationship between FAK and the PI3K/AKT signaling pathway in liver cancer. **I** The Cancer Genome Atlas (TCGA) analysis revealed the frequent overexpression of FAK in liver cancer tissues compared to normal tissues. **J** Kaplan–Meier analysis suggested a negative correlation between FAK expression level and the overall survival probability of liver cancer patients

The aforementioned results indicate that flavokawain C exhibits robust anti-proliferative and anti-migratory effects on liver cancer cells, prompting us to investigate its underlying anticancer mechanisms. RNA-seq was initially performed to assess the differential expression of genes and pathways between cells treated with flavokawain C and untreated cells. Simultaneously, the complete sequence data were deposited in the Gene Expression Omnibus (GEO) database with accession number GSE242009. The top 100 altered mRNA transcripts and their abundance are presented in Table S2. The changed genes were subsequently subjected to KEGG pathway enrichment analysis. Notably, the PI3K/AKT signaling pathway exhibited the most significant alterations among all signaling pathways, with focal adhesion ranking second (Fig. 7A). Furthermore, molecular docking studies revealed that flavokawain C formed five intermolecular hydrogen bonds with four amino acids of PI3K (Fig. 7B) and three hydrogen bonds with three amino acids of FAK (Fig. 7C). The molecular docking results were further validated according to the molecular dynamics (MD) simulation. The MD simulation data revealed that the root means square distance (RMSD) of the protein backbone of PI3K–FKC was converged after 3 ns of simulation and remained stable in the complete simulation run (Fig. 7D), and the protein backbone of FAK was converged after 1 ns of simulation and remained stable in the complete simulation run (Fig. 7E). In root-mean-square fluctuation (RMSF) analysis, the C and N terminal and the loop regions gave higher fluctuation in FAK-FKC and PI3K-FKC as shown in Fig. 7F and 7G. Collectively, these data suggest that flavokawain C could influence the PI3K/AKT and FAK signaling pathways by blocking the binding of PI3K and FAK.

The PI3K-AKT signaling pathway and focal adhesion were the most prominently altered pathways following flavokawain C treatment. The correlation between PI3K and FAK was further investigated by extracting transcriptome data from 835 liver carcinoma tissue biopsies from TCGA database. The results revealed a statistically significant positive correlation between the expressions of FAK and the PI3K/AKT signaling pathway (Fig. 7H). Moreover, bioinformatics analysis indicated that FAK was commonly overexpressed in liver cancer tissues compared to normal liver tissues (Fig. 7I). Patients with high FAK expression were correlated with a lower overall survival probability (Fig. 7J). These results strongly suggest that the FAK/PI3K/AKT signaling pathway plays a crucial role in the anticancer pharmacological activity of flavokawain C against liver cancer cells.

**Fig. 8:**
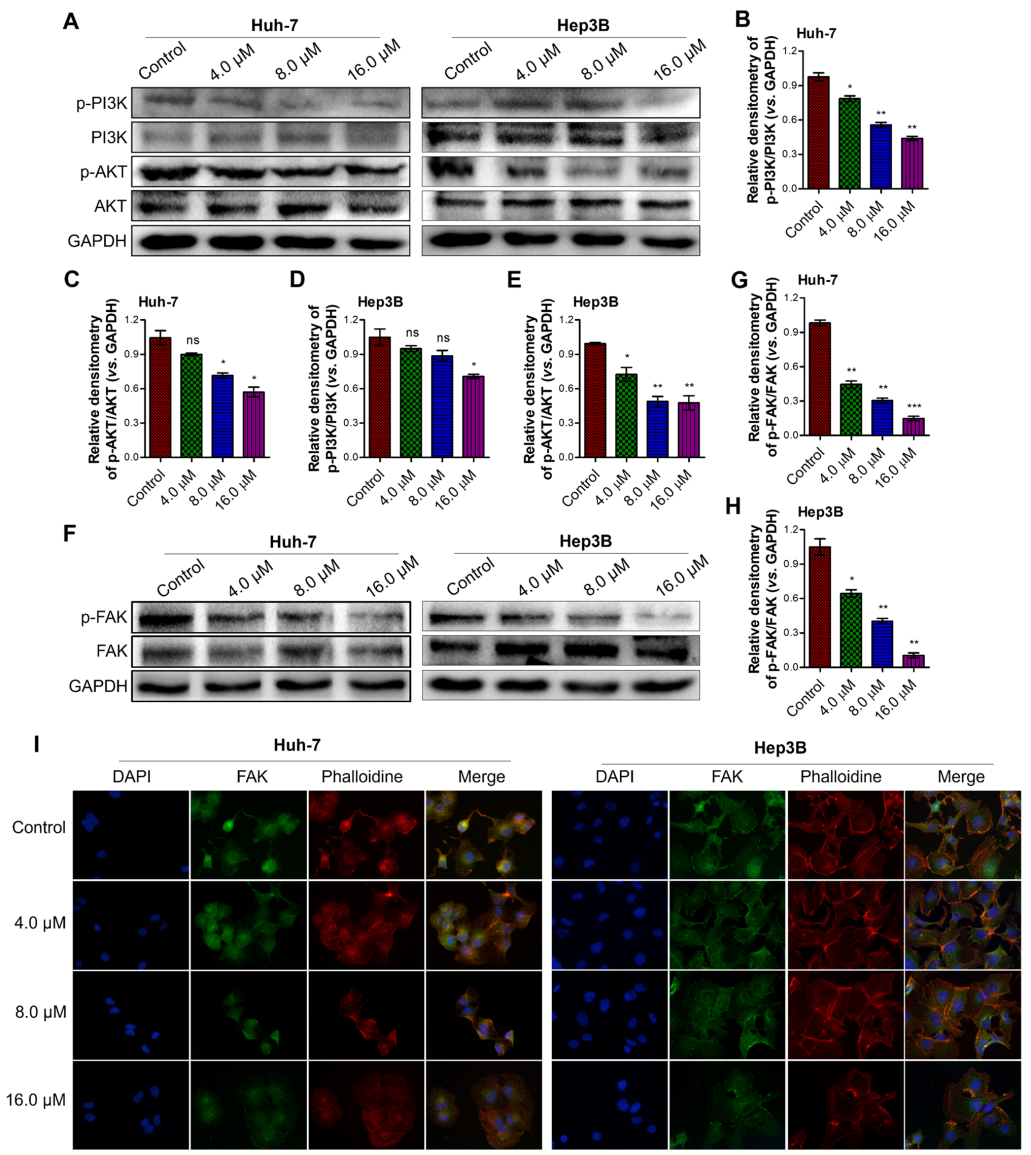
Flavokawain C inhibited the expression of p-PI3K, p-AKT and p-FAK, and reduces the front distribution of FAK in liver cancer cells. **A** Western blot assay determined the abundance of p-PI3K, PI3K, p-AKT and AKT in Huh-7 and Hep3B cells. Cells were treated with the indicated concentration of flavokavain C or 0.01% DMSO (Control) for 48 h before cell lysis. GAPDH was used as a positive control. **B–E** Quantitative analysis of A. **F** The same as A, except p-FAK and FAK protein were detected. **G**, **H** Quantitative analysis of F. **I** Representative images illustrating the reduction of FAK (green) distribution at the leading edge of cells following flavokawain C treatment, shown by immunofluorescence (IF) assay. Actin cytoskeleton was stained with phalloidin (red), and nuclei were stained with DAPI (blue). The results were obtained from three independent experiments and the values were presented as mean ± SD. The statistical analysis was conducted using a two-tailed, unpaired Student's *t*-test (ns means no significance with *p* > 0.05, *p < 0.05, **p < 0.01, ***p < 0.001)

Furthermore, the results from the western blot assay indicated that flavokawain C treatment inhibited the phosphorylation of FAK, PI3K, and AKT in both Huh-7 and Hep3B cells (Fig. 8A, F). Quantitative analysis revealed that flavokawain C inhibited the phosphorylation of FAK, PI3K, and AKT in a dose-dependent manner (Fig. 8B–E, G, H). Furthermore, the results from the IF assay suggested that, in addition to its inhibitory effect on p-FAK expression, flavokawain C treatment influenced the distribution of FAK in liver cancer Huh-7 and Hep3B cells (Fig. 8I).

### Flavokawain C suppressed Huh-7-derived xenograft tumor growth in mice

**Fig. 9:**
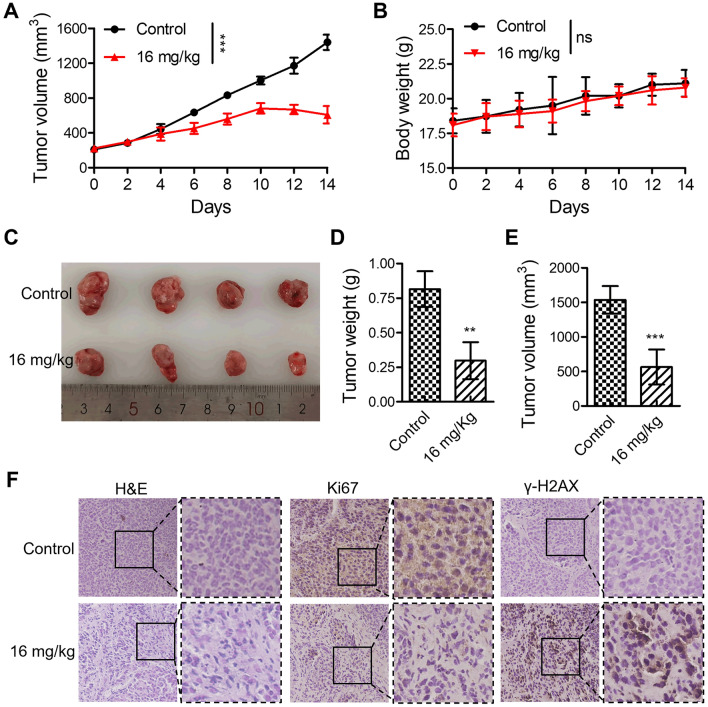
Flavokawain C inhibited the growth of Huh-7-derived xenograft tumor model in mice. **A** BALB/c nude mice bearing Huh-7-derived xenograft tumors were randomly divided into two groups and administered with either PBS or 16 mg/kg of flavokawain C every other day, and tumor volumes were measured over a two-week period. Tumor volumes were measured by a caliper and calculated by the formula Length × Width^2^/2. **B** Changes in body weight of nude mice treated with either PBS or flavokawain C (16 mg/kg) over the course of the administration. **C** Photographs of tumors taken after sacrificing the nude mice. **D**, **E** Quantitative analysis of the tumors mentioned in **C**. **F** Representative images of H&E staining and IHC staining for Ki67 and γ-H2AX on tumor samples. The results were obtained from three independent experiments and the values were presented as mean ± SD. The statistical analysis was conducted using a two-tailed, unpaired Student's *t*-test (ns means no significance with *p* > 0.05, **p < 0.01, ***p < 0.001)

On the base of flavokawain C exhibited moderate anti-proliferation effect on Huh-7 cells than Hep3B cells, BALB/c nude mice bearing Huh-7-derived xenograft tumors were randomly assigned to two groups and administered either PBS or 16 mg/kg of flavokawain C every other day. The results demonstrated that over the two-week administration period, flavokawain C significantly inhibited the tumor growth rate (Fig. 9A) without substantially affecting the mice's body weight (Fig. 9B). Photographs of tumors revealed smaller size, reduced weight, and diminished volume in the flavokawain C treatment group compared to the control group (Fig. 9C–E). Moreover, the results of H&E staining assay revealed that, in comparison with the control group, tumor tissues in the flavokawain C-treated group exhibited pronounced features such as cytoplasmic separation of the nucleus and nuclear fragmentation (Fig. 9F), suggesting cell death. Additionally, an IHC assay was employed to assess the levels of Ki67 and γ-H2AX, markers of proliferation and DNA damage, in tumor tissues with or without flavokawain C treatment. The results demonstrated a significant decrease in Ki67 levels in tumors treated with flavokawain C compared to PBS treatment (Fig. 9F), indicating that flavokawain C strongly inhibited tumor growth in mice. Furthermore, the results of the IHC assay revealed that, in comparison to the control group, tumor tissues treated with flavokawain C exhibited a significant increase in γ-H2AX expression levels (Fig. 9F), confirming that flavokawain C treatment induced substantial DNA damage. Taken together, these findings strongly suggest that flavokawain C effectively inhibits tumor growth in vivo.

## Discussion

Liver cancer, a substantial global health issue, has gained heightened attention due to its escalating incidence and elevated mortality rates (Anwanwan et al. 2020; Li et al. 2021). Chemotherapy and immunotherapy continue to serve as the primary therapeutic approaches for individuals with liver cancer (Anwanwan et al. 2020; Llovet et al. 2021). However, their efficacy is impeded by drug resistance, a limited response ratio, and adverse toxic effects (Anwanwan et al. 2020; Llovet et al. 2022; Sperandio et al. 2022). Thus, the imperative need arises for the exploration of novel therapeutic drugs and/or strategies to enhance clinical prognoses. Several studies have indicated that compared to synthetic small molecules, natural products might yield enhanced outcomes for cancer patients owing to diminished systemic toxicity (Anwanwan et al. 2020; Man et al. 2021). In this study, our results demonstrated that flavokawain C had stronger cytotoxicity on liver cancer cells than normal liver cells (Table S1), suggesting it maybe act as a potential drug for liver cancer treatment.

Targeting drugs specifically to tumor tissues is expected to enhance drug efficacy, utilization, and therapeutic outcomes, while also mitigating side effects (Zhao et al. 2020). Our findings illustrate that flavokawain C exhibits selective enrichment in liver tissue in vivo (Fig. 1A). This observation provides additional evidence supporting the potential role of flavokawain C as a novel targeted drug for liver cancer treatment. The proper functioning of the liver, which serves as the central hub for metabolism and detoxification, is crucial for maintaining overall health (Trefts et al. 2017). Furthermore, drug-induced liver injury poses a significant challenge in the treatment of liver cancer patients and can ultimately result in treatment failure (Katarey et al. 2016; Kumachev et al. 2021). Our findings indicated that treatment with flavokawain C does not alter the levels of ALT and AST in the serum, nor does it affect histomorphology (Fig. 1B–D). This suggests that the administration of flavokawain C is unlikely to cause liver injury. In aggregate, these findings contribute to the enhanced potential of utilizing flavokawain C in the clinical context of liver cancer treatment.

The FAK/PI3K/AKT signaling pathway is a pivotal player in cell proliferation, metastasis, and survival processes (Guo et al. 2020; Ke et al. 2022; Chen et al. 2023; Ye et al. 2023). Our findings indicate that flavokawain C is capable of downregulating the FAK/PI3K/AKT signaling pathway through the inhibition of FAK and PI3K phosphorylation (Fig. 8). Consequently, flavokawain C markedly suppressed the proliferation and migration of liver cancer cells in vitro and in vivo (Figs. 2A, 6, 9). It is well established that natural products often exhibit a tendency toward multi-target actions (Hashem et al. 2022). Therefore, the impact of flavokawain C on the FAK/PI3K/AKT signaling pathway may involve multiple molecules. In line with this, the results suggest that the reduction in p-FAK and p-PI3K proteins could stem from gene regulation due to flavokawain C treatment, as well as a direct interaction between flavokawain C and the ATP site of the FAK and/or PI3K protein (Fig. 7A–C).

In conclusion, this study has demonstrated that flavokawain C is specifically enriched in liver tissue and effectively inhibits the proliferation and migration of liver cancer cells by downregulating the FAK/PI3K/AKT signaling pathway. Importantly, this effect is achieved while causing minimal side effects on normal liver tissues, underscoring its potential as a novel liver cancer treatment drug with broad clinical applicability.

## Supplementary Information

Below is the link to the electronic supplementary material.

Supplementary file1 (DOCX 26 KB)

## Data Availability

Data will be made available on request.

## Abbreviations

ROS: Reactive oxygen species
ALT: Serum alanine transferase
AST: Serum aspartate aminotransferase
H&E: Hematoxylin eosin
IHC: Immunohistochemical
MTT: 3-(4,5)-Dimethylthiahiazo (-z-y1)-3,5-di-phenytetrazoliumromide
DMSO: Dimethyl sulfoxide
PI: Propidium iodide
IF: Immunofluorescence
TCGA: The Cancer Genome Atlas
FAK: Focal adhesion kinase
PI3K: Phosphoinositide 3-kinase
