# Supplementary material for: Flavokawain C inhibits proliferation and migration of liver cancer cells through FAK/PI3K/AKT signaling pathway
Source: J Cancer Res Clin Oncol. 2024 Mar 9;150(3):117. doi: 10.1007/s00432-024-05639-z (PMC10924746; doi:10.1007/s00432-024-05639-z)
Supplement: Supplementary file 1 — Supplementary file1 (DOCX 26 KB) [file 432_2024_5639_MOESM1_ESM.docx]

**Supporting information**

**Flavokawain C inhibits the proliferation and migration of liver cancer cells through FAK/PI3K/AKT signaling pathway**

Rong Wang^1,2^ · Rizhao Li^2^ · Huibing Yang^2^ · Xuejiao Chen^1,2^ · Lingliang Wu^2^ · Xiaohui Zheng^2^ · Yuepeng Jin^1^

^1^ National Key Clinical Specialty (General Surgery), The First Affiliated Hospital of Wenzhou Medical University, Wenzhou 325000, China

^2^ Wenzhou Medical University, Wenzhou 325000, China

**Correspondence:** Xiaohui Zheng (zhengxh@wmu.edu.cn), Yuepeng Jin (jinyuepeng@wzhospital.cn).

**Table S1.** *IC*_50_ values were determined by MTT assay.

| Compound | *IC*_50_ (μM) | | | |
| --- | --- | --- | --- | --- |
|  | MIHA | Huh-7 | Hep3B | HepG2 |
| Flavokawain C | 53.95 ± 5.08 | 23.42 ± 0.89 | 28.88 ± 2.60 | 30.71 ± 1.27 |

**Table S2.** Gene expression profile of flavokawain C-treated (**+**) (16 μM) and untreated (**-**) Huh-7 cells (list of top 100 changed genes)

| **GeneName** | **-** | **+** | **GeneName** | **-** | **+** | **GeneName** | **-** | **+** |
| --- | --- | --- | --- | --- | --- | --- | --- | --- |
| MATR3 | 0.24 | 7.63 | EFHD1 | 5.59 | 0.88 | PIGZ | 3.62 | 14.54 |
| BEST1 | 0.52 | 11.92 | TMEM59L | 2.29 | 0.37 | CA14 | 1.38 | 0.34 |
| YJEFN3 | 17.26 | 0.92 | H2BC5 | 1.4 | 8.14 | MUC13 | 0.96 | 3.84 |
| ACVR2B | 2.74 | 0.16 | NDUFC2-KCTD14 | 15.58 | 2.77 | MYLK3 | 0.6 | 0.15 |
| ENSG00000258790 | 4.58 | 0.27 | LYZ | 1.26 | 7.02 | CD68 | 0.5 | 1.99 |
| ADORA2A | 10.46 | 0.62 | DDC | 0.81 | 4.51 | ENSG00000286403 | 0.74 | 2.91 |
| ENSG00000285920 | 8.18 | 0.69 | SSC4D | 2.11 | 0.38 | PARM1 | 6.05 | 1.56 |
| ENSG00000285920 | 1.18 | 13.77 | RPL14P1 | 112.82 | 22.5 | LGR5 | 0.64 | 2.47 |
| KCNJ5 | 2.8 | 0.26 | ENSG00000285304 | 15.88 | 3.36 | KCNJ3 | 1.22 | 0.31 |
| EPHA3 | 13.55 | 1.35 | PPM1K | 1.14 | 0.25 | OR2A20P | 1.03 | 0.27 |
| FABP1 | 0.78 | 7.02 | CNTN4 | 0.58 | 0.12 | ADH6 | 0.61 | 2.31 |
| HABP2 | 1.22 | 10.91 | SAMD12 | 0.12 | 0.53 | COL2A1 | 1.53 | 0.4 |
| PDXP | 1.77 | 15.15 | NDRG1 | 2.81 | 12.74 | ERFE | 1.19 | 0.31 |
| ENSG00000283761 | 0.19 | 1.59 | CFH | 0.91 | 4.11 | DCHS1 | 5.44 | 1.44 |
| ENSG00000286001 | 0.38 | 2.85 | NID2 | 76.66 | 16.92 | MIR210HG | 1.35 | 5.11 |
| TMEM185A | 1.88 | 0.25 | SEMA6B | 5.11 | 1.13 | SSUH2 | 0.62 | 0.16 |
| ZNF254 | 1.34 | 0.18 | ST6GALNAC3 | 0.78 | 0.17 | PHYHIPL | 0.49 | 1.83 |
| DDTL | 0.88 | 6.26 | BSN | 0.27 | 0.06 | ARHGAP23 | 1.06 | 0.29 |
| LINC00304 | 0.38 | 2.66 | ZBED6 | 3.18 | 0.74 | DKK1 | 46.14 | 169.77 |
| ABCD2 | 1.42 | 0.2 | BCAS1 | 0.09 | 0.38 | SYNDIG1L | 2.2 | 0.6 |
| SMPD3 | 1.03 | 0.15 | CXCL8 | 0.61 | 2.59 | TENT5C | 1.04 | 0.28 |
| AGAP2-AS1 | 9.23 | 1.4 | GC | 11.75 | 49.59 | STC2 | 9.88 | 2.71 |
| ENSG00000288550 | 1.63 | 0.25 | SLC6A14 | 6.2 | 1.51 | PGC | 5.34 | 1.46 |
| ALPK3 | 1.55 | 0.24 | ARHGEF35-AS1 | 0.2 | 0.82 | FREM2 | 0.46 | 0.13 |
| CRLF1 | 6.59 | 1.04 | RGCC | 6.29 | 1.54 | ASNSP1 | 2.62 | 0.72 |
| OOEP | 2.29 | 0.63 | H2AC6 | 6.4 | 22.95 | OLFM3 | 2.94 | 0.82 |
| ENSG00000260257 | 0.51 | 1.82 | CAND2 | 1.98 | 0.55 | ALOX12B | 1.18 | 0.33 |
| GPC1 | 10.14 | 2.87 | GDA | 0.71 | 2.5 | FGF17 | 3.01 | 0.86 |
| BDNF | 1.01 | 0.29 | ENSG00000286185 | 2.6 | 0.75 | HJV | 2.81 | 0.82 |
| GABRA4 | 0.53 | 0.15 | DLG3 | 0.51 | 0.15 | ENSG00000226900 | 2.11 | 0.62 |
| LIMD1-AS1 | 0.73 | 0.21 | CLCNKA | 2.09 | 0.61 | ENSG00000214999 | 2.19 | 0.65 |
| SLC9A3-AS1 | 0.83 | 0.24 | COX20 | 1.76 | 5.93 | CLUHP3 | 0.53 | 1.79 |
| BAAT | 0.52 | 1.74 | IGDCC3 | 1.42 | 0.43 | METTL24 | 1.94 | 0.58 |
| ZNF114 | 2.46 | 0.74 |  |  |  |  |  |  |
